# Draft genome of extended-spectrum β-lactamase-producing multidrug-resistant *Salmonella enterica* serovar Brandearup strain DE0603 from an urban river in South Africa

**DOI:** 10.1128/mra.00957-25

**Published:** 2026-02-10

**Authors:** Sanelisiwe Thinasonke Duze, Musa Marimani, Mrudula Patel

**Affiliations:** 1 Department of Clinical Microbiology and Infectious Diseases, Faculty of Health Science, University of Witwatersrand 37708 , Johannesburg, South Africa; 2 Department of Anatomical Pathology, Faculty of Health Sciences, University of Witwatersrand 37708 , Johannesburg, South Africa; 3 Infection Laboratory Services Laboratory, National Health Laboratory Services 70685, Johannesburg, South Africa; California State University San Marcos, San Marcos, California, USA

**Keywords:** extended-spectrum β-lactamase-producing, *Salmonella*, Braenderup, multidrug resistance, Jukskie River

## Abstract

We report the draft genome of multidrug-resistant *Salmonella enterica* serovar Braenderup strain DE0603, isolated from an urban river in Johannesburg, South Africa. The isolate was identified as sequence type 22 and carries multiple resistance genes, including *blaCTX-_M-15_*, *qnrB19*, and a *parC* mutation (p.T57S; ACC→AGC) associated with fluoroquinolone resistance.

## ANNOUNCEMENT

Non-typhoidal *Salmonella* (NTS) infections pose a significant public health threat, particularly in sub-Saharan Africa (1–3). Exacerbating this challenge is the emergence of antimicrobial-resistant strains, particularly those resistant to fluoroquinolones and third-generation cephalosporins (3). Urban rivers can serve as environmental reservoirs that facilitate the transmission and persistence of multidrug-resistant NTS (4). This study reports the draft genome of an extended-spectrum β-lactamase (ESBL)-producing, multidrug-resistant (MDR) *Salmonella* Braenderup strain isolated from surface water.

Surface water samples from the Jukskei River (Johannesburg, South Africa) (5) were collected in sterile 2 L bottles, filtered through a 0.45 µm membrane, resuspended in 10 mL phosphate-buffered saline, and pre-enriched in 22.5 mL of ONE Broth-*Salmonella* (Thermo Fisher Scientific) at 42°C for 22 h. Cultures were streaked on Brilliance *Salmonella* agar and incubated overnight at 37°C. Presumptive colonies were confirmed using the Neg Combo 66 panel (Beckman Coulter) on a MicroScan WalkAway system, which provides species-level identification and antimicrobial susceptibility profile for gram-negative organisms. Genomic DNA was extracted from overnight 5% blood agar cultures using the QIAamp DNA Mini Kit (Qiagen) and quantified with NanoDrop 2000. Libraries were prepared with Illumina DNA Prep and sequenced on an Illumina NextSeq 2000 platform using a 2 × 150  bp paired-end protocol at 100× coverage. Adapter sequences were automatically removed during FASTQ generation by the Illumina NextSeq instrument, resulting in final reads with an average length of 143–144 bp. Illumina paired-end reads were analyzed using the JEKESA bioinformatics pipeline (v1.0, https://github.com/stanikae/jekesa), which incorporates multiple bioinformatics analysis tools (6), briefly described below with default parameters. Quality control and read filtering of raw paired-end reads were performed using FastQC v0.11.9 (7) and TrimGalore v0.6.7 (8). Reads were screened for contaminating sequences using Kraken2 against the MiniKraken2_v2_8GB database (9, 10). Species identification and closest reference detection were performed using BactInspector version 0.1.3 (11), and intra-species contamination was assessed using ConFindr (12). Reads were assembled using SPAdes v3.14.1 (13) and polished with Shovill v1.1.0 (14), followed by quality assessment with QUAST v5.2.0 (15). Annotation was performed using the NCBI Prokaryotic Genome Annotation Pipeline v6.10 (16). *Salmonella* serovar prediction was performed using SeqSero2 version 1.1.0 (http://denglab.info/SeqSero2) (17), SeqSero v1.2 (http://www.genomicepidemiology.org/services/) (18), and SISTR version 1.1.2 (https://github.com/phac-nml/sistr_cmd) (19). Multilocus sequence typing (MLST) based on PubMLST typing schemes (https://pubmlst.org/), antimicrobial resistance determinants, virulence factors, plasmids, and *Salmonella* pathogenicity islands were identified using tools on the Center for Genomic Epidemiology (CGE) platform (MLST v2.0, ResFinder v4.7.2, PlasmidFinder v2.1, and SPIFinder v2.0) with default parameters (20–25). The outputs from the JEKESA bioinformatics pipeline, as well as the CGE, are available at https://doi.org/10.6084/m9.figshare.30931334.

The isolate was identified as sequence type 22, a sequence type previously associated with a multi-country outbreak linked to contaminated melons in Europe (26, 27). The isolate carries multiple resistance genes, including *blaCTX-_M-15_* and *qnrB19* genes (Table 1). Moreover, the *mdsA* and *mdsB* genes associated with multidrug efflux pumps as well as a point mutation in the *parC* gene (p.T57S; ACC→AGC) associated with fluoroquinolone resistance were identified. The assembled genome had an average coverage of 100×, supporting the reliability of the point mutation, although site-specific coverage was not verified.

**TABLE 1:** Genome characteristics of the ESBL-producing MDR *Salmonella* Braenderup strain DE0603

Characteristics	Details
Isolation year	2023
Location	Gauteng, South Africa
Coordinates	25.98690°S; 27.99731°E
Number of reads	4,575,414
Number of contigs	45
Largest contig	1,412,055
N50	496,517
Genome length	4,913,321
G+C content	52%
Total genes	4,806
CDSs	4,590
tRNAs	74
rRNAs	5
MLST	ST22
Antibiotic resistance genes	*blaCTX-_M-15_*, *qnrB1*, *qnrB19*, *catA1*, *tetA*, *dfrA14*, *aac(6′)-Iaa*, *aadA1, mdsA*, and *mdsB*
Known chromosomal mutations	*parC* (p.T57S; ACC→AGC)
Phenotypic antibiotic-resistant profile^*a*^	Ampicillin, aztreonam, cefuroxime, cefotaxime, ceftazidime, cefepime, amikacin, and trimethoprim-sulfamethoxazole
Plasmids	Col(pHAD28), IncHI2, IncH12A
*Salmonella* pathogenicity islands	SPI-1, SPI-2, and SPI-14
Virulence genes	*iroB*, *iroC*, *sinH*
Stress response (heavy metals)	*golS*, *golT*, *pcoS*, *merD*, *merB*, *merR*, *arsC*, *terW*, *terZ*, and *terD*

^*a*^ Tested using the Neg Combo 66 panel on the MicroScan WalkAway system.

The presence of an ESBL-producing MDR *Salmonella* Braenderup strain DE0603 in this urban river raises serious public health concerns, as contaminated waterways can serve as reservoirs for antimicrobial resistance and potential sources of human infection.

## Data Availability

This Whole Genome Shotgun project has been deposited at DDBJ/ENA/GenBank under accession JBQJSD000000000. The version described in this paper is version JBQJSD000000000.1. The raw sequence reads (instrument FASTQs) were deposited in the NCBI under BioProject accession number PRJNA1304563 and SRA data accession number SRR35416639.
